# Chloroplast Translation Elongation Factor EF-Tu/SVR11 Is Involved in *var2*-Mediated Leaf Variegation and Leaf Development in *Arabidopsis*

**DOI:** 10.3389/fpls.2019.00295

**Published:** 2019-03-12

**Authors:** Siyu Liu, Lu Zheng, Jia Jia, Jia Guo, Mengdi Zheng, Jun Zhao, Jingxia Shao, Xiayan Liu, Lijun An, Fei Yu, Yafei Qi

**Affiliations:** State Key Laboratory of Crop Stress Biology for Arid Areas and College of Life Sciences, Northwest A&F University, Yangling, China

**Keywords:** EF-Tu, chloroplast development, leaf variegation, retrograde signal, VAR2

## Abstract

Chloroplasts are semiautonomous organelles, retaining their own genomes and gene expression apparatuses but controlled by nucleus genome encoded protein factors during evolution. To analyze the genetic regulatory network of FtsH-mediated chloroplast development in *Arabidopsis*, a set of suppressor mutants of *yellow variegated* (*var2*) have been identified. In this research, we reported the identification of another new *var2* suppressor locus, *SUPPRESSOR OF VARIEGATION11* (*SVR11*), which encodes a putative chloroplast-localized prokaryotic type translation elongation factor EF-Tu. *SVR11* is likely essential to chloroplast development and plant survival. *GUS* activity reveals that *SVR11* is abundant in the juvenile leaf tissue, lateral roots, and root tips. Interestingly, we found that *SVR11* and *SVR9* together regulate leaf development, including leaf margin development and cotyledon venation patterns. These findings reinforce the notion that chloroplast translation state triggers retrograde signals regulate not only chloroplast development but also leaf development.

## Introduction

Chloroplasts are essential organelles for eukaryotic photosynthetic species, enabling the chemical reactions powered by light energy to reduce CO_2_ to carbohydrates. It is believed that chloroplasts evolved from ancient prokaryotic cyanobacteria through endosymbiosis (Martin et al., 2002). This co-evolution process, especially the transfer of most genes of chloroplast progenitors to the host nuclear genomes, have given rise to modern-day chloroplast genomes with only around 120 genes, in contrast to the more than 3,000 genes of the current genome of cyanobacteria, such as *Synechocystis* sp. (Timmis et al., 2004). The physical separation of nuclear and chloroplast genomes raises at least two important implications. First, the remaining genes in chloroplast genomes are expressed with prokaryotic gene expression systems, which are regulated by the nuclear genome and must respond to developmental and environmental conditions (Jarvis and López-Juez, 2013). Second, many photosynthetic protein complexes are chimeric in nature, and are composed of subunits encoded by both nuclear and plastid genomes, and intricate regulation at different levels are necessary for the optimal assembly of these complexes. The semi-autonomous nature of the chloroplast thus necessitates a fine coordination between the two genomes (Kleine and Leister, 2016).

Higher plants have evolved multiple strategies to facilitate the expression and coupling of nuclear and chloroplast genomes. At the translation level, chloroplasts utilize a prokaryotic translation system featuring the 70S ribosome (Yamaguchi and Subramanian, 2000; Yamaguchi et al., 2000; Tiller and Bock, 2014). Prokaryotic translation initiates through the binding of 30S ribosomal subunit to the Shine-Dalgarno sequence of mRNA, and the subsequent association of initiator tRNA leads to the formation of pre-initiation complex, and this process is assisted by initiation factors IFs (Laursen et al., 2005). In *Arabidopsis*, chloroplast IF1 and IF2 homologs are encoded by the nuclear genome, and null mutant alleles of *Arabidopsis* IF1 and IF2 are embryonic lethal, indicating they are essential genes for plant viability (Miura et al., 2007; Shen et al., 2013; Nesbit et al., 2015). Further recruitment of the 50S ribosomal subunit to pre-initiation complex forms an active initiation complex (Laursen et al., 2005). The translation process requires elongation factors EF-Tu, EF-G, and EF-Ts to incorporate aminoacyl-tRNAs into 70S ribosomes (Krab and Parmeggiani, 2002). EF-Tu is a prokaryotic elongation factor belonging to the GTP-binding protein family (Krab and Parmeggiani, 2002). During translation elongation, GTP-bound EF-Tu forms a ternary complex with aminoacyl-tRNA to facilitate the transport of cognate aminoacyl-tRNA to the A-site of the 70S ribosome. Next, the innate GTPase activity of EF-Tu hydrolyzes the GTP to GDP, and GDP-bound EF-Tu is released from ribosome and recycled to GTP-bound EF-Tu mediated by EF-Ts for the next round of elongation (Krab and Parmeggiani, 1998). During endosymbiosis, genes coding for many of the chloroplast 70S ribosomal proteins and most translational factors have been transferred to the nuclear genome and are subject to nuclear regulation. Partial loss of chloroplast EF-Tu activities in *Arabidopsis*, maize, and tomato reduced heat tolerance, suggesting chloroplast EF-Tu is involved in the plant response to environmental changes (Ristic et al., 2004; Li et al., 2018).

Chloroplast gene expression is also regulated by post-translational mechanisms including the operation of a vast array of protease systems (Nishimura et al., 2016). An intriguing group of proteolytic enzymes that has attracted attention is the chloroplast FtsH proteases, due to the unique leaf variegation phenotypes of *yellow variegated1* (*var1*) and *yellow variegated2* (*var2*) mutants, defective in thylakoid-localized FtsH proteins AtFtsH5 and AtFtsH2, respectively (Chen et al., 2000; Takechi et al., 2000; Sakamoto et al., 2002). FtsH proteins belong to the AAA (ATPase associated with various cellular activities) ATPase superfamily, which is ubiquitously present in prokaryotes and eukaryotes, as well as in mitochondria and chloroplasts (Janska et al., 2013). Thylakoid FtsH complexes comprise four members of FtsH proteins, FtsH1 and FtsH5 (type A) and FtsH2 and FtsH8 (type B), in which VAR2/AtFtsH2 is one of the most abundant subunits (Yu et al., 2004; Zaltsman et al., 2005; Nishimura et al., 2016). Biochemical analysis suggested that thylakoid FtsHs are required to degrade photo-damaged reaction center protein D1 during Photosystem II repair cycle (Lindahl et al., 2000; Kato et al., 2009; Malnoë et al., 2014). Interestingly, VAR2/AtFtsH2-mediated post-translational regulation is closely related with chloroplast translation. Multiple genetic screens for *var2* suppressors in several laboratories have yielded an increasing number of genetic factors involved in chloroplast transcription, translation and post-translational turnover (Park and Rodermel, 2004; Yu et al., 2008; Adam et al., 2011; reviewed in Liu et al., 2010 and Putarjunan et al., 2013). Recently, we reported a new *var2* suppressor mutant, *svr9-1*, which is defective in a bona fide chloroplast-localized prokaryotic translation initiation factor IF3 (Zheng et al., 2016). In Bacteria, initiation factor IF3, encoded by the essential *infC* gene, binds to the 30S ribosomal subunit to promote dissociation of the 70S ribosome for ribosome recycling and translation initiation (Laursen et al., 2005). Down regulation of *SVR9*, alone or with its homologous gene *SVR9L1*, not only suppresses *var2* leaf variegation phenotype, but also causes leaf developmental abnormalities including serrated leaf margin and altered cotyledon venation patterns (Zheng et al., 2016). The characterization of *var2* suppressor genes thus provides a unique opportunity to uncover additional regulators of chloroplast translation.

Here, we report the identification of a new *var2* suppressor mutant, *svr11-1*. Molecular cloning, complementation and protein localization studies confirmed that *SVR11* encodes a putative prokaryotic translation elongation factor EF-Tu, which is localized in chloroplasts. Interestingly, functional genetic analysis of *SVR11*, *SVR9*, and *SVR9L1* showed that *svr11-1 svr9-1* double mutants display a more serrated leaf margin and altered cotyledon venation patterns compared to those of the wild type, while *svr11-1 svr9-1 svr9-1l-1*/*+* mutants have an even more pronounced leaf serration. These data suggest that chloroplast translation elongation factor EF-Tu/SVR11 not only regulate chloroplast development, but also act synergistically with chloroplast translation initiation factor IF3/SVR9 to dictate leaf margin and cotyledon vascular development. Our findings uncover a new translation elongation factor in regulating chloroplast and leaf development in *Arabidopsis*.

## Materials and Methods

### Plant Materials and Growth Conditions

*Arabidopsis thaliana* plants used in this study are all in the Columbia-0 background. The T-DNA insertion line *CS819179* (*svr11-3*) was obtained from the *Arabidopsis* Biological Resource Center (ABRC); the accurate position of each T-DNA insertion sites were identified by sequencing PCR products that include plant genomic DNA and T-DNA left border sequences. *Arabidopsis* seeds were grown at 22°C under continuous illumination (∼100 μmol m^−2^s^−1^) on commercial soil mix (Pindstrup, Denmark). All seeds were stratified for 2 days at 4°C before sown on soil or half strength MS medium. For heat stress at a moderate level, 8-day-old seedlings were treated at 38°C for 90 min, and then moved into 22°C for recovery (Queitsch et al., 2000).

### Chlorophyll Fluorescence Imaging

Chlorophyll fluorescence was measured with 2-week-old plants using Open FluorCam FC800-O (Photon Systems Instruments; Czechia). Whole plants were dark adapted for 10 min to oxidize the plastoquinone pool before measurement, and the minimum fluorescence *F*_O_ was measured. The maximum fluorescence *F*_M_ was determined by a saturating flash of light. The maximum quantum yield of photosystem II (*F*_V_/*F*_M_) is calculated as *F*_M_ – *F*_O_/*F*_M_. Measurement of *F*_V_/*F*_M_ was performed in three independent biological repeats.

### RNA Manipulations, Vector Constructions, and Transformations

Total RNAs were extracted using Trizol RNA reagent (Life Technologies, Carlsbad, CA, United States) according to the manufacturer’s instructions. For semi-quantitative RT-PCR analysis, first-strand cDNA was synthesized from 1 μg DNase-treated total RNA using a PrimeScript reverse transcription kit (Roche, Switzerland). The gene-specific primers used in this study are listed in Table 1. The semi-quantitative RT-PCR was performed in three independent biological repeats.

**Table 1:** Primers used in this study.

Primer name	Primer sequences	Notes
20360 F	5′-CATGGATCCACCCTAGCTTCTCGATTTCTC-3′	*p35S:: intronSVR11*
20360 R	5′-CATGGATCCGAAAGCAAGTAGAGATGCTCAC-3′	
27700 inF	5′-CATCTCGAGACTCTCGCTTTCTTCATCATCTC-3′	*p35S:: intronSVR11*
27700 inR	5′-CATTCTAGAGCTTTGAAAGAGTAAACGAGTCC-3′	
20360 GFPR	5′-CATGGGATCCACCACCACCACCACCTTGAGGATCGTCCCAATAAC-3′	*p35S:: SVR11-GFP*
02930 F	5′-CGCGGATCCATGGCGTCCGTTGTTCTTCG-3′	*p35S:: SVR11-like-GFP*
02930 GFPR	5′-CGCGGATCCGGTCATCACTTTTGATACAAC-3′	
20360 PF	5′-CATTCTAGACTACCCTTTTGCTGTCTTGTAAG-3′	*pSVR11::uidA*
20360 PR	5′-CATGGATCCGAAGATGGAATTGGAGAGCAGAG-3′	
20360 F3	5′-GTTACGATTTGTGACGTGTG-3′	Genotyping
20360 F1	5′-ACCCTAGCTTCTCGATTTCTC-3′	
20360 R1:	5′-GAAAGCAAGTAGAGATGCTCAC-3′	
20360 R2:	5′-CAGCTAAAGCCTCATCAAGAATC-3′	
CM35E	5′-AAGATGCCTCTGCCGACAGT-3′	Sequencing
pCB308R	5′-AACGACAATCTGAGCTCCAC-3′	Genotyping
uidA-R	5′-GTTCAGTTCGTTGTTCACAC-3′	Genotyping
SKC12	5′-TTGACAGTGACGACAAATCG-3′	Inverse PCR
OCS3	5′-TAGAGCTCTTATACTCGAGG-3′	Inverse PCR

To complement the *svr11-1* and *049-002* mutants, a full-length At4g20360 (*SVR11*) cDNA was amplified by Primer STAR^TM^ HS DNA polymerase (Takara) using primers 20360F and 20360R. The PCR product was digested with *Bam*HI and cloned into the *Bam*HI site of pBluescript KS+. The sequenced *SVR11* fragment was then transferred into pBI111L-intron plasmid which is modified from pBI111L plasmid (Yu et al., 2004). In brief, the first intron sequence of At5g27700 at its 5′ UTR region were amplified with primers 27700inF (*X*baI) and 27700inR (*Bam*HI), and inserted into the multiple cloning site of pBI111L as a chimeric intron at the 5′ UTR region of insertion genes. The resulting construct was transformed into Agrobacterium by electroporation. *Arabidopsis* transformation was performed as described (Clough and Bent, 1998).

### Inverse PCR

Genomic DNA extracted from *049-002* homozygous plants was digested with restriction enzyme *Eco*RI overnight. The DNA fragments were further precipitated with 2.5 volumes of ethanol and 0.1 volumes of 3M sodium acetate (pH 5.2). After dissolving in Milli-Q water, DNA fragments were ligated with T4 DNA Ligase. Inverse PCR were performed with Pfu DNA Polymerase using primers SKC12 and OCS3. One 1.5 kb PCR amplified band was sequenced with the SKC12 primer.

### Evolutionary Analysis

Full-length protein sequences of SVR11 homologous proteins from dicots *Arabidopsis thaliana*, monocots *Oryza sativa*, moss *Physcomitrella patens*, green algae *Chlamydomonas reinhardtii*, yeast *Saccharomyces cerevisiae*, and prokaryotic species such as *Synechocystis* and *Escherichia coli* were obtained from NCBI using the BLASTP program. Evolutionary analyses were conducted in MEGA X, and the Neighbor-joining algorithm was used to generate the initial tree (Kumar et al., 2018). The accession numbers of protein sequences were included.

### Transient Expression of SVR11-GFP and SVR11-Like-GFP

In order to generate a C-terminal GFP-tagged SVR11, the coding sequences of SVR11 amplified with primers 20360F and 20360 GFPR, SVR11-like with 02930F and 02930 GFPR, were cloned into transient expression vector pTF486. The resulting construct were designated *p35S::SVR11-GFP* and *p35S::SVR11-like-GFP*. *Arabidopsis* leaf protoplast preparation and transient expression of GFP constructs were performed as described by Yoo et al. (2007). Bright field images and fluorescent signals from GFP and chlorophyll autofluorescence were monitored using a Leica DM5000B fluorescent microscope (Leica, Germany).

### Histochemical GUS Staining

Amplified with primers 20360 PF and 20360 PR, a 1,493-bp genomic DNA fragment upstream of the start codon of At4g20360 was cloned into pCB308 (Xiang et al., 1999), to generate *SVR11* promoter-β-glucuronidase (GUS) construct *pSVR11::uidA*. The construct *pSVR11::uidA* was transformed into wild-type *Arabidopsis* plant, and *pSVR11::uidA* lines were screened with BASTA. GUS activities were assayed in the T2 generations (Jefferson et al., 1987). The GUS staining was performed with three independent transgenic lines.

### Leaf Silhouettes and Cotyledon Veins Observation

For leaf silhouettes imaging, individual true leaves were covered with water and photographed using Research stereo microscope (SMZ25; Nikon) equipped with a CCD camera (DS-U3; Nikon). Those photos were converted to black and white by Adobe Photoshop 8.0.1 by filled the leaf blades with black, so that it is easier to observe the silhouettes of leaves between different phenotypes. The leaf dissection index was calculated as described (perimeter^2^/4π^∗^leaf area, Bilsborough et al., 2011). For cotyledon veins observation, cotyledons of 10-day-old plants were cut down and decolorized in 70% ethanol, till the cotyledons blade turned colorless without chlorophyll and the veins become clearly visible then the cotyledon samples were photographed by stereo microscope photographing. Cotyledon vein patterns were quantified in three independent biological replicates with tested lines containing at least 100 seedlings each time.

## Results

### Identification of *049-002* and *svr11-1*

**FIGURE 1:**
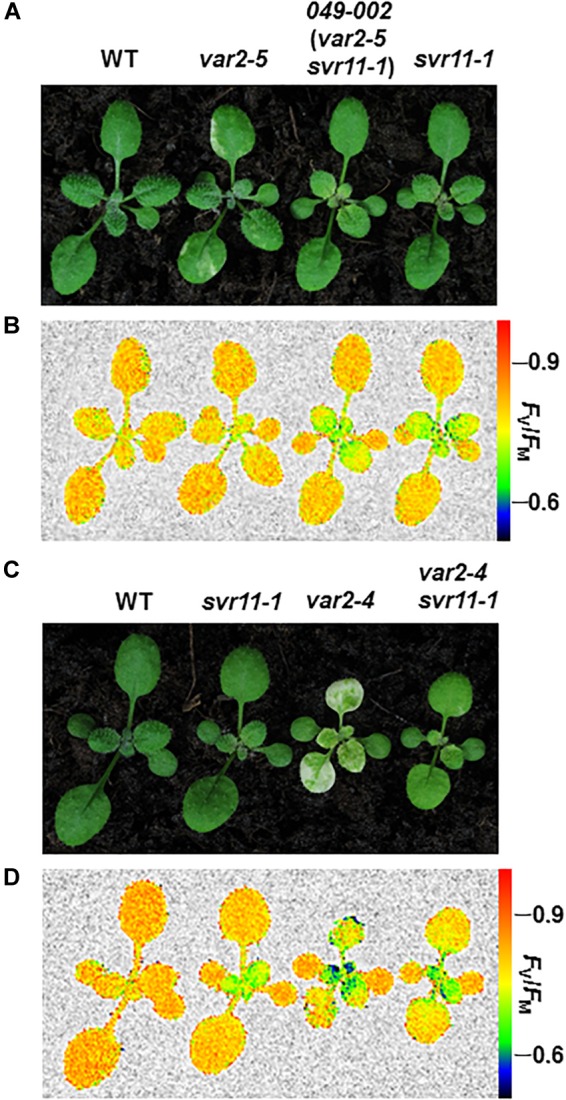
Phenotypes of single and double suppressor mutants. **(A)** Representative 2-week-old seedlings of wild type (WT), *var2-5*, double suppressor mutant *049-002* (*var2-5 svr11-1*), and *svr11-1* plants. **(C)** Representative 2-week-old seedlings of wild type (WT), *svr11-1*, *var2-4*, and the double mutant *var2-4 svr11-1*. **(B,D)** Representative *F*_V_/*F*_M_ measurement of the same group of plants used in **(A,C)**, respectively. The false color scale representing for the value of *F*_V_/*F*_M_ is at the right of the figure. The measurement of *F*_V_/*F*_M_ was repeated three times.

We have performed extensive genetic suppressor screens for mutants that could reverse the *var2* leaf variegation phenotype (reviewed in Liu et al., 2010 and Putarjunan et al., 2013). Here, we report the identification of a new recessive suppressor line, designated as *049-002*, from the activation tagging T-DNA mutant population in the *var2-5* mutant background (Yu et al., 2008). Following our naming sequence, the suppressor gene locus was named as *SUPPRESSOR OF VARIEGATION11* (*SVR11*) and the mutant allele in *049-002* as *svr11-1*. Overall, *049-002* (*var2-5 svr11-1*) did not show the characteristic leaf variegation phenotype of *var2-5*, indicating that s*vr11-1* is a robust suppressor of *var2-5* (Figure 1A). In addition, the statures of *049-002* and *svr11-1* resembled that of wild type, suggesting overall plant growth was not dramatically altered by the *svr11-1* mutation. Interestingly, both *049-002* and *svr11-1* showed a virescent phenotype, i.e., a gradual yellow to green leaf color gradient along the leaf proximal-distal axis (Figure 1A). This virescence phenotype was correlated with a reduction of photosynthetic parameters, as indicated by the *F*_V_/*F*_M_ (the maximum quantum yield of photosystem II) of whole plant chlorophyll fluorescence imaging (Figure 1B). *svr11-1* could also reverse the leaf variegation of the *var2-4* mutant, a stronger allele of *var2*, indicating that the suppression of *var2* leaf variegation by *svr11-1* does not depend on the nature of *var2* mutation and is not allele specific (Figure 1C,D).

### Molecular Cloning of *SVR11*

**FIGURE 2:**
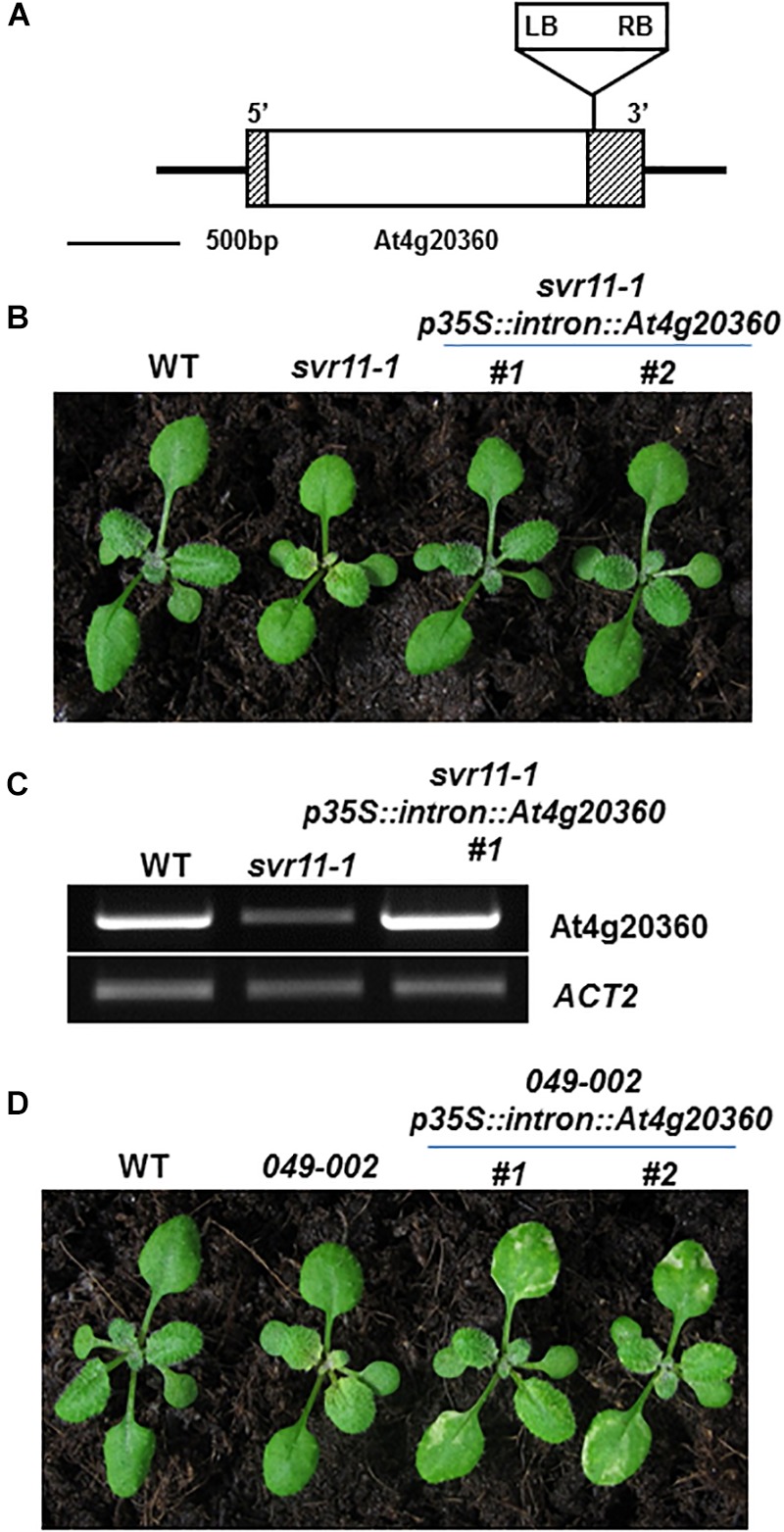
Cloning of *SVR11* and complementation of *svr11-1*. **(A)** A T-DNA insertion was identified in the 3′-UTR of At4g20360 by inverse PCR. The left border (LB) and the right border (RB) were indicated. Gray boxes and the white box represent UTRs and the exon, respectively. **(B)** Representative 2-week-old wild-type, *svr11-1* and *svr11-1 p35S::intron At4g20360* (constitutive expression line of *At4g20360* in *svr11-1* background). **(C)** The accumulation of At4g20360 transcripts was indicated by semi-quantitative RT-PCR. The semi-quantitative RT-PCR was repeated three times. **(D)** Representative 2-week-old wild-type, *049-002* and *049-002 p35S::intron At4g20360* (constitutive expression of *At4g20360* in *049-002* background).

To clone *SVR11*, we first determined that the *svr11-1* mutant phenotype co-segregated with the resistance to herbicide Basta, suggesting the mutant is tagged by T-DNA insert(s) (data not shown). Next, we carried out inverse PCR to identify the T-DNA insertion site and sequencing of inverse PCR products confirmed that the T-DNA was inserted in the 3′ UTR of *At4g20360* (Figure 2A). We found that *At4g20360* expression was reduced in *svr11-1*, likely a consequence of T-DNA insertion in 3′ UTR (Figure 2C). Complementation analysis was executed to confirm that the virescent phenotype in *svr11-1* and the suppression of *var2* variegation in *049-002* were due to the disruption of *SVR11* expression. To this end, we generated a binary vector in which a full-length *At4g20360* cDNA was driven by the constitutive CaMV 35S promoter. In addition, sequences of the first intron of At5g27700 were placed between the 35S promoter and the cDNA sequences to achieve better expression (Rose et al., 2008). This construct (p35S::intron::At4g20360) was transformed into *svr11-1* and *049-002*, respectively. We recovered multiple independent transgenic lines and confirmed that elevated expression of At4g20360 was able to complement the virescent phenotype of *svr11-1* (Figure 2B). Furthermore, ectopic expression of At4g20360 was able to restore the *var2-5* leaf variegation phenotype in *049-002* background (Figure 2D). Together, these data indicate that the virescent chloroplast defect in *svr11-1* and the suppression of *var2* leaf variegation in *049-002* were caused by reduced expression of At4g20360, and that *At4g20360* is *SVR11*.

### SVR11/At4g20360 Defines a Putative Prokaryotic EF-Tu in Chloroplasts

**FIGURE 3:**
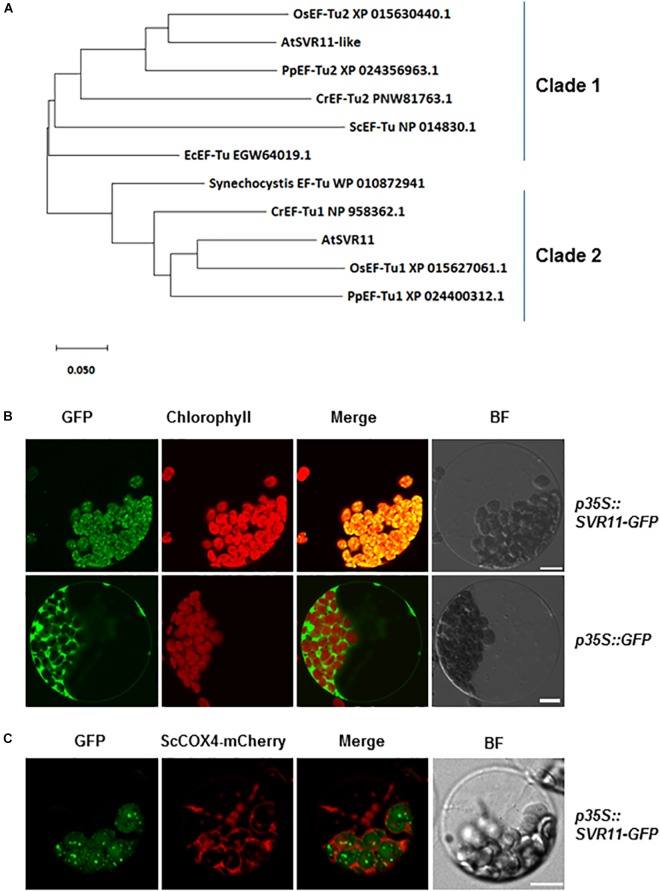
Evolutionary relationships of SVR11 homologs and subcellular localization of SVR11. **(A)** Phylogenetic analysis of the SVR11 protein family. Full-length protein sequences of SVR11 homologous proteins of different species indicated by the accession numbers were obtained from NCBI using BLASTP. The neighbor-joining algorithm was used to generate the initial tree. **(B)** Leaf protoplasts prepared from wild type *Arabidopsis* plants were transformed with the *p35S::SVR11-GFP* fusion construct or the control construct p35S::GFP. Confocal microscopy was used to monitor fluorescence signals from the GFP channel (500–550 nm) and chlorophyll autofluorescence (663–738 nm). Bright field (BF) images served as controls for protoplast integrity. **(C)** Transient expression of *p35S::SVR11-GFP* fusion protein in protoplasts isolated from plants expressing a mitochondrion marker ScCOX4-mCherry (570–620 nm). Representative images of a single protoplast are shown. Bar stands for 10 μm.

Homologous sequences of SVR11 from different species were obtained from National Center for Biotechnology Information (NCBI) and their evolutionary relationship was analyzed (Figure 3A). SVR11 and SVR11-like proteins from these species were all annotated as prokaryotic translation elongation factor EF-Tu homologs. In prokaryotic organisms such as cyanobacteria and *E. coli*, only one copy of EF-Tu was identified. In contrast, eukaryotic photosynthetic species such as *Arabidopsis*, rice, moss, and green algae contains at least two EF-Tu homologs (Figure 3A).

The EF-Tu sequences grouped in the same clade with SVR11 in the phylogenetic tree were all predicted to contain chloroplast transit peptides (Figure 3A Clade 2, Emanuelsson et al., 1999). To confirm the sub-cellular localizations of SVR11, a transient expression vector was generated expressing a full-length *SVR11* cDNA fused in-frame at its C-terminus with green fluorescent protein (GFP), under the control of the 35S promoter (*p35S::SVR11-GFP*). This construct, as well as a control vector containing only the GFP (*p35S::GFP*), were introduced into Wild-type *Arabidopsis* leaf protoplasts, respectively, and their expressions were monitored by confocal microscopy. Figure 3B shows that GFP signals for *p35S::SVR11-GFP* appeared as distinct foci, which overlapped nicely with chlorophyll auto-fluorescence signals, suggesting co-localizations with chloroplasts. To examine if SVR11-GFP could also be targeted to mitochondria, we transient expressed *p35S::SVR11-GFP* in protoplasts isolated from transgenic lines stably expressing a mitochondrion marker protein tagged with mCherry, ScCOX4-mCherry (Nelson et al., 2007). SVR11-GFP did not overlap with signals of ScCOX4-mCherry, suggesting SVR11-GFP is likely not targeted to mitochondria (Figure 3C). These results demonstrate that the SVR11-GFP is targeted into the chloroplast and SVR11 is a nuclear encoded chloroplast protein. SVR11-like was predicted to be a mitochondrial EF-Tu (Nikolovski et al., 2012), or identified in the mitochondrial soluble protein by mass spectrometry (Ito et al., 2006). Interestingly, SVR11-like-GFP aggregate to large or small dots in the cytosol, neither targeted into mitochondria nor to chloroplasts (Supplementary Figure S1).

### Moderate Heat Stress Has Little Impact on *var2* Variegations

It was reported that knock-down of chloroplastic EF-Tu in maize, *Arabidopsis*, and tomato mutants reduced heat tolerance (Ristic et al., 2004; Li et al., 2018). We then test if heat stress can affect the variegation phenotype of *var2* mutants. To avoid lethality caused by the severe heat stress at 45°C, a moderate level of heat stress at 38°C for 90 min were used as suggested (Queitsch et al., 2000). Moderate heat stress had little impact either on the variegation phenotype of *var2* and *var2* background suppressor mutants, or on the virescent phenotype of *svr11-1* (Supplementary Figure S2).

### SVR11 Is Essential to Plant Development

**FIGURE 4:**
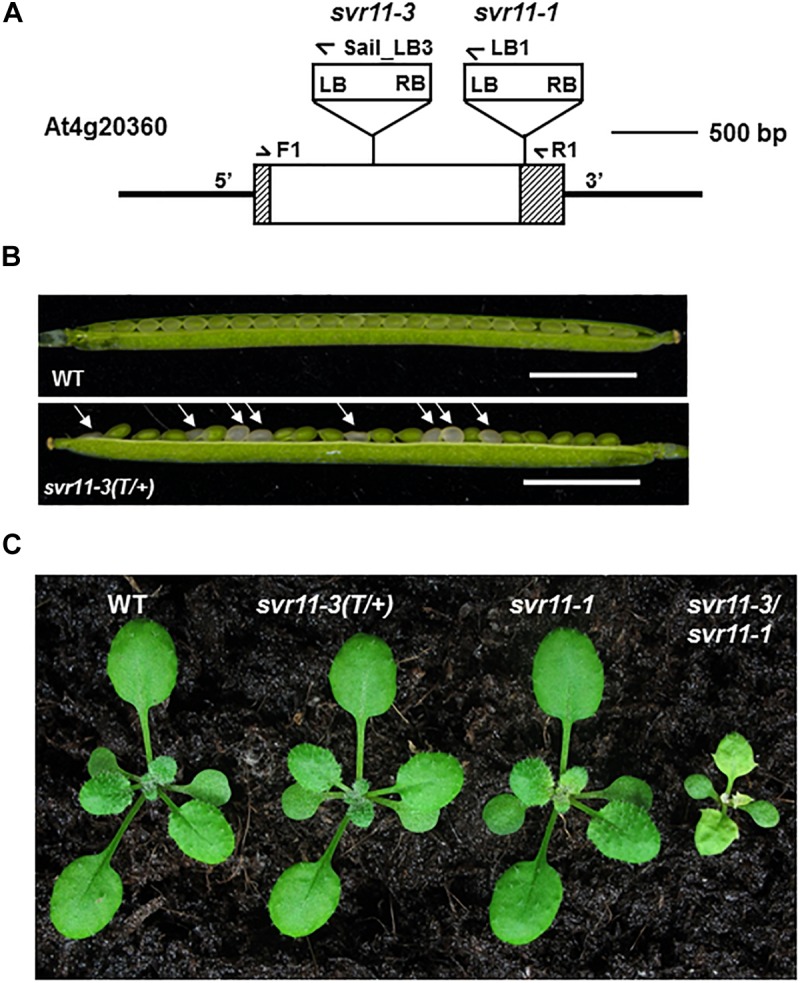
Phenotypic characterization of the *svr11* T-DNA insertion null allele mutant. **(A)** Identification of a null allele of *SVR11, svr11-3*. **(B)** Developing seeds within a silique from a heterozygous *svr11-3* mutant and wild-type at the same developing state. Bar stands for 2,500 μm. **(C)** Representative 2-week-old wild-type, *svr11-3(T/+)*, *svr11-1*, *svr11-3/svr11-1* plants.

To further examine the roles that SVR11 play in plant development, we sought for loss-of-function alleles of *SVR11*. We obtained a second allele of *svr11*, *CS819179* which contained a T-DNA inserted in the encoding sequence of *SVR11* (Figure 4A). No homozygous T-DNA insertion line was identified even backcrossed to wild-type five times, probably due to homozygote is embryo lethal. Terminated ovules were observed in the developing siliques in heterozygous mutants (Figure 4B). We renamed *CS819179* as *svr11-3*, and then crossed heterozygous *svr11-3(T/+)* to *svr11-1*. *svr11-1*/*svr11-3* was obtained by genotyping F1 generation, which is much smaller in size than *svr11-1* and the leaf blade is yellow-colored. We speculated that the phenotypic defect severity was determined by *SVR11* damage degree (Figure 4C).

### SVR11 Is Abundant in Juvenile Tissues

**FIGURE 5:**
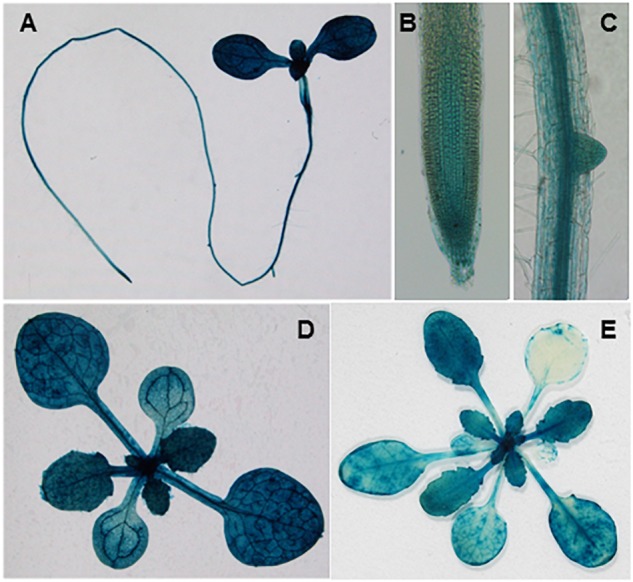
Tissue expression patterns of *SVR11*. GUS staining of transgenic plants expressing the *SVR11* promoter-GUS fusion vector *pSVR11::uidA*. The GUS staining was performed with three independent transgenic lines. Representative whole seedling **(A)**, root tip **(B)**, and lateral root **(C)** of 6-day-old transgenic seedling grown on half-strength Murashige and Skoog (MS) solid medium containing 1% sucrose. Representative leaf tissues above the soil from 2-week-old **(D)** and 3-week-old **(E)** plants.

To characterize the spatial and temporal expression profiles of *SVR11*, we generated a fusion construct in which the β-glucuronidase gene (GUS) gene was controlled by the *SVR11* promoter (1.5-kb region upstream of the *SVR11* start codon). This vector was transformed into wild-type plants and GUS activities of *pSVR11:GUS* transgenic lines were assayed at different growth stages. In brief, in 6-day-old seedlings GUS expression was detected including in the root tip and lateral roots, suggesting that SVR11 activities are necessary for both photosynthetic and non-photosynthetic tissues (Figure 5A–C), also in 2-week-old plants and 3-week-old juvenile rosette leaves (Figure 5D,E).

### Chloroplast EF-Tu and IF3 Regulate Leaf Development

**FIGURE 6:**
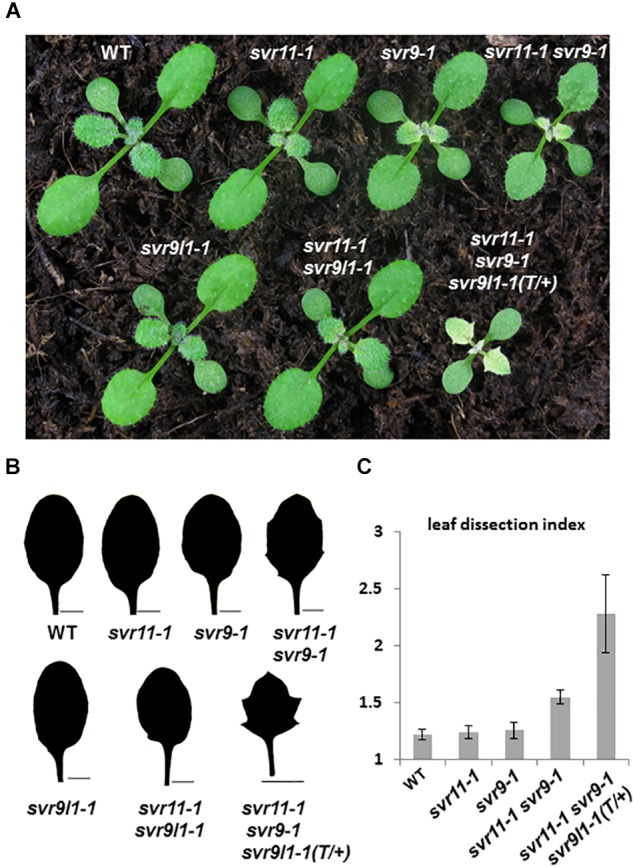
Genetic interaction between *SVR11* and *SVR9*. **(A)** Representative 12-day-old wild-type, *svr11-1*, *svr9-1*, *svr11-1svr9-1*, *svr9l1-1*, *svr11-1 svr9l1-1*, and *svr11-1 svr9-1 svr9l1-1(T/+)*. **(B)** The third leaf silhouettes of 20-day-old plants. Bar for 2,000 μm. **(C)** Quantitative comparisons of leaf shapes in WT, *svr11-1*, *svr9-1*, *svr11-1 svr9-1*, and *svr11-1 svr9-1svr9l1-1(T/+)* shown in **(B)**, based on the leaf dissection index (perimeter^2^/4π^∗^leaf area). Fifteen individuals of each genotype were used in the statistical analysis.

Previously, we have reported a *var2* suppressor locus *SVR9*, encoding a chloroplast translation initiation factor IF3, which mediates *var2* leaf variegation and leaf marginal serration formation (Zheng et al., 2016). Next, we tested genetically the functional relationships between *SVR11* and *SVR9*, as well as *SVR9L1*, a functionally redundant homolog of *SVR9* (Figure 6) (Zheng et al., 2016). At the single mutant level, *svr9-1* showed a stronger degree of virescence than that of *svr11-1*, and *svr9l1-1* showed a WT-like phenotype, as reported (Figure 6A) (Zheng et al., 2016). Consistent with both EF-Tu and IF3’s involvement in translation, *svr11-1 svr9-1* mutants were more virescent (Figure 6). The virescent level of *svr11-1*, *svr9-1* and *svr11-1 svr9-1* double mutants were quantified by measurement of *F*_V_/*F*_M_ (Supplementary Figure S3). Interestingly, *svr11-1 svr9-1*, but not *svr11-1 svr9l1-1*, showed a prominent leaf margin serration phenotype (Figure 6B). Furthermore, we obtained mutants that are homozygous for *svr11-1* and *svr9-1* while heterozygous for *svr9l1-1* (*svr11-1 svr9-1 svr9l1-1* T/+). These mutants not only showed strong virescence phenotype, the leaf serration was also the most conspicuous (Figure 6B,C). The leaf serrations were further quantified by the leaf dissection index (perimeter^2^/4π^∗^leaf area) (Bilsborough et al., 2011) (Figure 6C).

**FIGURE 7:**
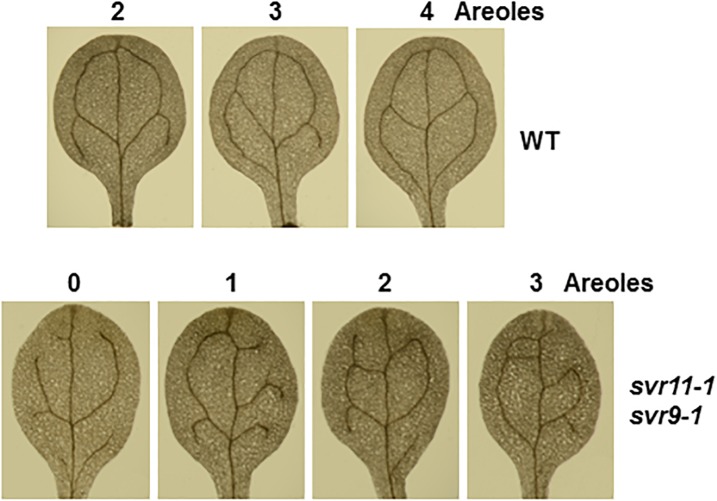
Representative 10-day-old cotyledon vein patterns with different numbers of areoles from wild-type and *svr11-1 svr9-1*.

We have shown that leaf serration phenotype may be associated with leaf vasculature development (Zheng et al., 2016). We then tested leaf vascular development and examined the cotyledon venation patterns in *svr11-1* and *svr11-1 svr9-1* double mutant. The numbers of closed areoles in mature cotyledons are indicators of leaf vascular development (Sieburth, 1999), and cotyledons from 10-day-old seedlings were observed under a dissecting microscope. Wild type cotyledons with two, three, and four areoles were predominant (Zheng et al., 2016, and also in the research, Table 2). In *svr11-1*, although the similar percentage of cotyledons show two, three or four areoles compared to wild type, cotyledons with only one areole were also identified in *svr11-1* (Table 2). Noticeably, cotyledons with only one areole or no closed areoles were drastically increased in *svr11-1 svr9-1* (Figure 7 and Table 2). Taken together, these data show that chloroplast translation EF-Tu and IF3 activities act synergistically to regulate leaf margin and cotyledon vascular development.

**Table 2:** Quantification of cotyledon vein patterns in wild type, *svr11-1* and *svr11-1 svr9-1*.

Genotype	Total	Zero areole	One areole	Two areoles	Three areoles	Four areoles	Five areoles
WT	353	N.A.	N.A.	171 (48.4%)	134 (38.0%)	48 (13.6%)	N.A.
*svr11-1*	432	N.A.	21 (4.8%)	194 (44.9%)	167 (38.7%)	50 (11.6%)	N.A.
*svr11-1 svr9-1*	345	56 (16.2%)	147 (42.6%)	105 (30.4%)	32 (9.3%)	5 (1.4%)	N.A.

*Cotyledon vein patterns were quantified in three independent biological replicates with each genotype line containing at least 100 seedlings. Numbers were added together from three independent biological replicates. Percentages of different types of areoles are indicated in the parentheses. N.A., not applicable.*

## Discussion

Chloroplasts are semi-autonomous organelles that derived from ancient cyanobacterium-like organisms through the process of endosymbiosis (Nowack and Weber, 2018). One of the key evidence supporting the endosymbiosis theory is the discovery of prokaryotic gene expression systems in the chloroplast. In this study, we identified *svr11-1* as a genetic suppressor of the *Arabidopsis var2* mutant and confirmed that *SVR11* codes for a chloroplast-localized prokaryotic translation elongation factor EF-Tu. As a prokaryotic elongation factor, GTP-bound EF-Tu facilitates the transport of aminoacyl-tRNA to the A-site of the 70S ribosome during translation elongation (Krab and Parmeggiani, 2002). Consistent with their critical roles in translation, null alleles or higher-order mutants of many translation factors are lethal, suggesting essential roles of these factors for plant survival (Miura et al., 2007; Bryant et al., 2011; Zheng et al., 2016). Interestingly, *svr11-1* mutant is phenotypically reminiscent of *svr9-1*, defective in a chloroplast translation initiation factor IF3 (Zheng et al., 2016). Both mutants show a distinct virescent phenotype with more pronounced reductions of chlorophyll accumulation and photosynthetic capacities at young, dividing tissues, suggesting their activities are required at early process (Lopez-Juez and Pyke, 2005). This is in agreement with the higher expression of these genes in young tissues and higher needs for their activities in those tissues. It is conceivable that a higher demand for translation capacities at younger stage of development is necessary to establish robust phototropic growth. Findings from our group and others have shown that mutations in chloroplast 70S ribosome proteins mostly lead to rather uniform pale green or pale yellow leaf colorations (Bryant et al., 2011; Romani et al., 2012; Tiller et al., 2012; Liu et al., 2013). These findings suggest a possible regulatory way for translation factors and ribosomal proteins during chloroplast and leaf development at early stages.

Thylakoid-localized FtsHs are ATP-dependent zinc metalloproteases participating in the degradation of damaged photosynthetic subunits, especially damaged PSII reaction center D1 subunits during photoinhibition (Nishimura et al., 2016). *Arabidopsis* mutants *var1* and *var2*, defective in thylakoid-localized FtsH proteins VAR1/AtFtsH5 and VAR2/AtFtsH2, respectively, show unique leaf variegation phenotypes, suggesting that these FtsHs may play additional roles in chloroplast development besides D1 degradation (Chen et al., 2000; Takechi et al., 2000). Molecular genetics analyses of *var2* suppressor mutants have also established that the chloroplast development defect, i.e., the leaf variegation phenotype, of *var2* is dependent on functional chloroplast gene expression, especially chloroplast translation, providing further support for additional roles of VAR2/AtFtsH2 in chloroplast development (Miura et al., 2007; Yu et al., 2008; Liu et al., 2010, 2013; Putarjunan et al., 2013). Recently, we reported two *var2* suppressor loci, *SVR10*, coding for a member of circularly permuted GTPase family involved in the processing of plastid ribosomal RNAs, and *SVR9*, a chloroplast translation initiation factor IF3 involved in chloroplast translation (Kim et al., 2012; Qi et al., 2016; Zheng et al., 2016). In this report, building on the *var2* suppressor screening work, we found that a mutation in *SVR11*, encoding a chloroplast translation elongation factor EF-Tu, can suppress *var2* leaf variegated phenotype. The identification of *SVR11* as a *var2* suppression locus on one hand strengthens the functional relationship between VAR2/AtFtsH2 and chloroplast translation, on the other hand, it also provides further indication that VAR2/AtFtsH2 is related to the chloroplast translation process in general, rather than a specific functional link with individual components. Interestingly, through genetic enhancer analysis, we have recently for the first time established an intriguing link between VAR2/AtFtsH2 and cytosolic translation (Wang et al., 2018). In contrary to chloroplast translation, the reduction of cytosolic translation dramatically enhances *var2* leaf variegation, suggesting chloroplast development in *var2* is intimately regulated by cytosolic translation. Based on the suppression by reduced chloroplast translation and the enhancement by reduced cytosolic translation, a model was proposed in which FtsH may serve as an important factor in mediating the balance of cytosolic and chloroplast translation (Wang et al., 2018). Although the molecular mechanism underlying the maintenance of this balance remains unclear in higher plants, nuclear and mitochondrial translation balance has been shown to be vital of protein homeostasis in other model organisms (Topf et al., 2016).

The coordination between the nuclear and the chloroplast genome and gene expression requires fine regulation of bi-directional communications from nucleus to chloroplasts (anterograde) and also from chloroplasts to the nucleus (retrograde) (Jarvis and López-Juez, 2013). Given the importance and complexity, it came as no surprise that multiple regulatory pathways have been uncovered to ensure the coupling of the genomes. Canonical work have used the expressions of nuclear encoded photosynthetic genes, for example *LhcB* or *RbcS*, as marker genes to probe the retrograde regulation of these genes when chloroplast functional states were disturbed, for instance, when treated with photo-bleaching herbicide norflurazon or chloroplast translation inhibitor lincomycin (Nott et al., 2006; Chi et al., 2013; Kleine and Leister, 2016). It has also been long recognized that the retrograde regulation of nuclear gene expression also involves the modulation of leaf development by the functional state of chloroplasts (Pogson et al., 2015). For example, abnormal leaf mesophyll developments were observed in the white leaf sectors of *Arabidopsis immutans* mutant, or in white tissues after norflurazon treatment (Aluru et al., 2009). Despite the accumulating evidence, our understanding of how states of chloroplasts affect leaf development remains limited. We previously reported that the chloroplast translation initiation factor IF3/SVR9 regulates chloroplast development, as well as leaf development, including leaf margin and cotyledon vasculature development (Zheng et al., 2016). In addition, we reported that mutations in *SVR9* affect auxin homeostasis, and leaf margin development in a CUC2-dependent way (Nikovics et al., 2006; Zheng et al., 2016). In this work, we found that *SVR11* also regulate leaf margin and cotyledon venation. Moreover, *SVR11* and *SVR9* work synergistically to regulate leaf margin development and cotyledon venation patterns. These findings reinforce the notion that chloroplast translation defects can trigger a signaling pathway to regulate leaf development (Zheng et al., 2016). This pathway seems to be activated only by certain types of translation defects caused mainly by the lack of translation factors, as not all chloroplast translation mutant display related phenotypes. Recently, it was shown that VAR2/AtFtsH2 may mediate a singlet oxygen signaling pathway from chloroplasts to the nucleus (Wang et al., 2016). Future research is warranted to address the relationship between these pathways and the components of this signaling pathway from the chloroplast to the nucleus.

## Author Contributions

YQ and FY conceived and coordinated the study and wrote the manuscript. SL, LZ, JJ, JG, and MZ designed, performed, and analyzed the experiments shown in Figure 1–7. JZ, JS, LA, and XL provided technical assistance and contributed to the preparation of the figures. All authors reviewed the results and approved the final version of the manuscript.

## Conflict of Interest Statement

The authors declare that the research was conducted in the absence of any commercial or financial relationships that could be construed as a potential conflict of interest.

## Supplementary Material

FIGURE S1 Transient expression of *p35S::SVR11-like-GFP* fusion protein in leaf protoplasts of plants expressing the mitochondrion marker ScCOX4-mCherry. Representative images of a single protoplast are shown. Bar stands for 10 μm.

FIGURE S2 Representative seedlings treated with a moderate level heat stress. Eight-day-old seedlings grown at 22°C were treated at 38°C for 90 min, and then returned into 22°C for recovery. After 6 days recovery, seedlings were photographed.

FIGURE S3 Representative 2-week-old seedlings and the corresponding *F*_V_/*F*_M_ images of wild type, *svr11-1*, *svr9-1*, and the double mutant *svr11-1 svr9-1*. The average value of *F*_O_, *F*_M_, *F*_V_/*F*_M_ were calculated from at least 10 individuals from each genotype.

## Abbreviations

CaMV: Cauliflower Mosaic Virus
EF-Ts: elongation factor thermo stable
EF-Tu: elongation factor thermo unstable
FtsH: filamentous temperature sensitive H
GFP: green fluorescence protein
*SVR*: *SUPPRESSOR OF VARIEGATION;* UTR, untranslated region
*var2*: *yellow variegated2.*

## References

[B1] Adam Z. Frottin F. Espagne C. Meinnel T. Giglione C. (2011). Interplay between N-terminal methionine excision and FtsH protease is essential for normal chloroplast development and function in Arabidopsis. *Plant Cell* 23 3745–3760. 10.1105/tpc.111.087239 22010036 PMC3229147

[B2] Aluru M. R. Zola J. Foudree A. Rodermel S. R. (2009). Chloroplast photooxidation-induced transcriptome reprogramming in Arabidopsis immutans white leaf sectors. *Plant Physiol.* 150 904–923. 10.1104/pp.109.135780 19386811 PMC2689989

[B3] Bilsborough G. D. Runions A. Barkoulas M. Jenkins H. W. Hasson A. Galinha C. (2011). Model for the regulation of Arabidopsis thaliana leaf margin development. *Proc. Natl. Acad. Sci. U.S.A.* 108 3424–3429. 10.1073/pnas.1015162108 21300866 PMC3044365

[B4] Bryant N. Lloyd J. Sweeney C. Myouga F. Meinke D. (2011). Identification of nuclear genes encoding chloroplast-localized proteins required for embryo development in Arabidopsis. *Plant Physiol.* 155 1678–1689. 10.1104/pp.110.168120 21139083 PMC3091104

[B5] Chen M. Choi Y. Voytas D. F. Rodermel S. (2000). Mutations in the Arabidopsis VAR2 locus cause leaf variegation due to the loss of a chloroplast FtsH protease. *Plant J.* 22 303–313. 10.1046/j.1365-313x.2000.00738.x 10849347

[B6] Chi W. Sun X. Zhang L. (2013). Intracellular signaling from plastid to nucleus. *Annu. Rev. Plant Biol.* 64 559–582. 10.1146/annurev-arplant-050312-120147 23394498

[B7] Clough S. J. Bent A. F. (1998). Floral dip: a simplified method for Agrobacterium-mediated transformation of *Arabidopsis thaliana*. *Plant J.* 16 735–743. 10.1046/j.1365-313x.1998.00343.x 10069079

[B8] Emanuelsson O. Nielsen H. von Heijne G. (1999). ChloroP, a neural network-based method for predicting chloroplast transit peptides and their cleavage sites. *Protein Sci.* 8 978–984. 10.1110/ps.8.5.978 10338008 PMC2144330

[B9] Ito J. Heazlewood J. L. Millar A. H. (2006). Analysis of the soluble ATP-binding proteome of plant mitochondria identifies new proteins and nucleotide triphosphate interactions within the matrix. *J. Proteome Res.* 5 3459–3469. 10.1021/pr060403j 17137349

[B10] Janska H. Kwasniak M. Szczepanowska J. (2013). Protein quality control in organelles – AAA/FtsH story. *Biochim. Biophys. Acta* 1833 381–387. 10.1016/j.bbamcr.2012.03.016 22498346

[B11] Jarvis P. López-Juez E. (2013). Biogenesis and homeostasis of chloroplasts and other plastids. *Nat. Rev. Mol. Cell Biol.* 14 787–802. 10.1038/nrm3702 24263360

[B12] Jefferson R. A. Kavanagh T. A. Bevan M. W. (1987). GUS fusions: beta-glucuronidase as a sensitive and versatile gene fusion marker in higher plants. *EMBO J.* 6 3901–3907. 10.1002/j.1460-2075.1987.tb02730.x 3327686 PMC553867

[B13] Kato Y. Miura E. Ido K. Ifuku K. Sakamoto W. (2009). The variegated mutants lacking chloroplastic FtsHs are defective in D1 degradation and accumulate reactive oxygen species. *Plant Physiol.* 151 1790–1801. 10.1104/pp.109.146589 19767385 PMC2785964

[B14] Kim B. H. Malec P. Waloszek A. von Arnim A. G. (2012). Arabidopsis BPG2: a phytochrome-regulated gene whose protein product binds to plastid ribosomal RNAs. *Planta* 236 677–690. 10.1007/s00425-012-1638-6 22526496

[B15] Kleine T. Leister D. (2016). Retrograde signaling: organelles go networking. *Biochim. Biophys. Acta* 1857 1313–1325. 10.1016/j.bbabio.2016.03.017 26997501

[B16] Krab I. M. Parmeggiani A. (1998). EF-Tu, a GTPase odyssey. *Biochim. Biophys. Acta* 1443 1–22. 10.1016/S0167-4781(98)00169-9 9838020

[B17] Krab I. M. Parmeggiani A. (2002). Mechanisms of EF-Tu, a pioneer GTPase. *Prog. Nucleic Acid Res. Mol. Biol.* 71 513–551. 10.1016/S0079-6603(02)71050-7 12102560

[B18] Kumar S. Stecher G. Li M. Knyaz C. Tamura K. (2018). MEGA X: molecular evolutionary genetics analysis across computing platforms. *Mol. Biol. Evol.* 35 1547–1549. 10.1093/molbev/msy096 29722887 PMC5967553

[B19] Laursen B. S. Sørensen H. P. Mortensen K. K. Sperling-Petersen H. U. (2005). Initiation of protein synthesis in bacteria. *Microbiol. Mol. Biol. Rev.* 69 101–123. 10.1128/MMBR.69.1.101-123.2005 15755955 PMC1082788

[B20] Li X. Cai C. Wang Z. Fan B. Zhu C. Chen Z. (2018). Plastid translation elongation factor tu is prone to heat-induced aggregation despite its critical role in plant heat tolerance. *Plant Physiol.* 176 3027–3045. 10.1104/pp.17.01672 29444814 PMC5884619

[B21] Lindahl M. Spetea C. Hundal T. Oppenheim A. B. Adam Z. Andersson B. (2000). The thylakoid FtsH protease plays a role in the light-induced turnover of the photosystem II D1 protein. *Plant Cell* 12 419–431. 10.1105/tpc.12.3.419 10715327 PMC139841

[B22] Liu X. Rodermel S. R. Yu F. (2010). A var2 leaf variegation suppressor locus, SUPPRESSOR OF VARIEGATION3, encodes a putative chloroplast translation elongation factor that is important for chloroplast development in the cold. *BMC Plant Biol.* 10:287. 10.1186/1471-2229-10-287 21187014 PMC3022910

[B23] Liu X. Zheng M. Wang R. Wang R. An L. Rodermel S. R. (2013). Genetic interactions reveal that specific defects of chloroplast translation are associated with the suppression of var2-mediated leaf variegation. *J. Integr. Plant Biol.* 55 979–993. 10.1111/jipb.12078 23721655

[B24] Lopez-Juez E. Pyke K. A. (2005). Plastids unleashed: their development and their integration in plant development. *Int. J. Dev. Biol.* 49 557–577. 10.1387/ijdb.051997el 16096965

[B25] Malnoë A. Wang F. Girard-Bascou J. Wollman F. A. de Vitry C. (2014). Thylakoid FtsH protease contributes to photosystem II and cytochrome b6f remodeling in *Chlamydomonas reinhardtii* under stress conditions. *Plant Cell* 26 373–390. 10.1105/tpc.113.120113 24449688 PMC3963582

[B26] Martin W. Rujan T. Richly E. Hansen A. Cornelsen S. Lins T. (2002). Evolutionary analysis of Arabidopsis, cyanobacterial, and chloroplast genomes reveals plastid phylogeny and thousands of cyanobacterial genes in the nucleus. *Proc. Natl. Acad. Sci. U.S.A.* 99 12246–12251. 10.1073/pnas.182432999 12218172 PMC129430

[B27] Miura E. Kato Y. Matsushima R. Albrecht V. Laalami S. Sakamoto W. (2007). The balance between protein synthesis and degradation in chloroplasts determines leaf variegation in Arabidopsis yellow variegated mutants. *Plant Cell* 19 1313–1328. 10.1105/tpc.106.049270 17416734 PMC1913758

[B28] Nelson B. K. Cai X. Nebenführ A. (2007). A multicolored set of *in vivo* organelle markers for co-localization studies in Arabidopsis and other plants. *Plant J.* 51 1126–1136. 10.1111/j.1365-313X.2007.03212.x 17666025

[B29] Nesbit A. D. Whippo C. Hangarter R. P. Kehoe D. M. (2015). Translation initiation factor 3 families: what are their roles in regulating cyanobacterial and chloroplast gene expression? *Photosynth. Res.* 126 147–159. 10.1007/s11120-015-0074-4 25630975

[B30] Nikolovski N. Rubtsov D. Segura M. P. Miles G. P. Stevens T. J. Dunkley T. P. (2012). Putative glycosyltransferases and other plant Golgi apparatus proteins are revealed by LOPIT proteomics. *Plant Physiol.* 160 1037–1051. 10.1104/pp.112.204263 22923678 PMC3461528

[B31] Nikovics K. Blein T. Peaucelle A. Ishida T. Morin H. Aida M. (2006). The balance between the MIR164A and CUC2 genes controls leaf margin serration in Arabidopsis. *Plant Cell* 18 2929–2945. 10.1105/tpc.106.045617 17098808 PMC1693934

[B32] Nishimura K. Kato Y. Sakamoto W. (2016). Chloroplast proteases: updates on proteolysis within and across suborganellar compartments. *Plant Physiol.* 171 2280–2293. 10.1104/pp.16.00330 27288365 PMC4972267

[B33] Nott A. Jung H. S. Koussevitzky S. Chory J. (2006). Plastid-to-nucleus retrograde signaling. *Annu. Rev. Plant Biol.* 57 739–759. 10.1146/annurev.arplant.57.032905.105310 16669780

[B34] Nowack E. C. M. Weber A. P. M. (2018). Genomics-informed insights into endosymbiotic organelle evolution in photosynthetic eukaryotes. *Annu. Rev. Plant Biol.* 69 51–84. 10.1146/annurev-arplant-042817-040209 29489396

[B35] Park S. Rodermel S. R. (2004). Mutations in ClpC2/Hsp100 suppress the requirement for FtsH in thylakoid membrane biogenesis. *Proc. Natl. Acad. Sci. U.S.A.* 101 12765–12770. 10.1073/pnas.0402764101 15304652 PMC515127

[B36] Pogson B. J. Ganguly D. Albrecht-Borth V. (2015). Insights into chloroplast biogenesis and development. *Biochim. Biophys. Acta* 1847 1017–1024. 10.1016/j.bbabio.2015.02.003 25667967

[B37] Putarjunan A. Liu X. Nolan T. Yu F. Rodermel S. (2013). Understanding chloroplast biogenesis using second-site suppressors of immutans and var2. *Photosynth. Res.* 116 437–453. 10.1007/s11120-013-9855-9 23703455

[B38] Qi Y. Zhao J. An R. Zhang J. Liang S. Shao J. (2016). Mutations in circularly permuted GTPase family genes AtNOA1/RIF1/SVR10 and BPG2 suppress var2-mediated leaf variegation in Arabidopsis thaliana. *Photosynth. Res.* 127 355–367. 10.1007/s11120-015-0195-9 26435530

[B39] Queitsch C. Hong S. W. Vierling E. Lindquist S. (2000). Heat shock protein 101 plays a crucial role in thermotolerance in Arabidopsis. *Plant Cell* 12 479–492. 10.1105/tpc.12.4.479 10760238 PMC139847

[B40] Ristic Z. Wilson K. Nelsen C. Momcilovic I. Kobayashi S. Meeley R. (2004). A maize mutant with decreased capacity to accumulate chloroplast protein synthesis elongation factor (EF-Tu) displays reduced tolerance to heat stress. *Plant Sci.* 167 1367–1374. 10.1016/j.plantsci.2004.07.016

[B41] Romani I. Tadini L. Rossi F. Masiero S. Pribil M. Jahns P. (2012). Versatile roles of Arabidopsis plastid ribosomal proteins in plant growth and development. *Plant J.* 72 922–934. 10.1111/tpj.12000 22900828

[B42] Rose A. B. Elfersi T. Parra G. Korf I. (2008). Promoter-proximal introns in Arabidopsis thaliana are enriched in dispersed signals that elevate gene expression. *Plant Cell* 20 543–551. 10.1105/tpc.107.057190 18319396 PMC2329928

[B43] Sakamoto W. Tamura T. Hanba-Tomita Y. Murata M. Sodmergen (2002). The VAR1 locus of Arabidopsis encodes a chloroplastic FtsH and is responsible for leaf variegation in the mutant alleles. *Genes Cells* 7 769–780. 10.1046/j.1365-2443.2002.00558.x 12167156

[B44] Shen Y. Li C. McCarty D. R. Meeley R. Tan B. C. (2013). Embryo defective12 encodes the plastid initiation factor 3 and is essential for embryogenesis in maize. *Plant J.* 74 792–804. 10.1111/tpj.12161 23451851

[B45] Sieburth L. E. (1999). Auxin is required for leaf vein pattern in Arabidopsis. *Plant Physiol.* 121 1179–1190. 10.1104/pp.121.4.1179 10594105 PMC59485

[B46] Takechi K. Sodmergen Murata M. Motoyoshi F. Sakamoto W. (2000). The YELLOW VARIEGATED (VAR2) locus encodes a homologue of FtsH, an ATP-dependent protease in Arabidopsis. *Plant Cell Physiol.* 41 1334–1346. 10.1093/pcp/pcd067 11134419

[B47] Tiller N. Bock R. (2014). The translational apparatus of plastids and its role in plant development. *Mol Plant.* 7 1105–1120. 10.1093/mp/ssu022 24589494 PMC4086613

[B48] Tiller N. Weingartner M. Thiele W. Maximova E. Schöttler M. A. Bock R. (2012). The plastid-specific ribosomal proteins of Arabidopsis thaliana can be divided into non-essential proteins and genuine ribosomal proteins. *Plant J.* 69 302–316. 10.1111/j.1365-313X.2011.04791.x 21923745

[B49] Timmis J. N. Ayliffe M. A. Huang C. Y. Martin W. (2004). Endosymbiotic gene transfer: organelle genomes forge eukaryotic chromosomes. *Nat. Rev. Genet.* 5 123–135. 10.1038/nrg1271 14735123

[B50] Topf U. Wrobel L. Chacinska A. (2016). Chatty mitochondria: keeping balance in cellular protein homeostasis. *Trends Cell Biol.* 26 577–586. 10.1016/j.tcb.2016.03.002 27004699

[B51] Wang L. Kim C. Xu X. Piskurewicz U. Dogra V. Singh S. (2016). Singlet oxygen- and EXECUTER1-mediated signaling is initiated in grana margins and depends on the protease FtsH2. *Proc. Natl. Acad. Sci. U.S.A.* 113 E3792–E3800. 10.1073/pnas.1603562113 27303039 PMC4932964

[B52] Wang R. Zhao J. Jia M. Xu N. Liang S. Shao J. (2018). Balance between cytosolic and chloroplast translation affects leaf variegation. *Plant Physiol.* 176 804–818. 10.1104/pp.17.00673 29142022 PMC5761769

[B53] Xiang C. Han P. Lutziger I. Wang K. Oliver D. J. (1999). A mini binary vector series for plant transformation. *Plant Mol. Biol.* 40 711–717. 10.1023/a:1006201910593 10480394

[B54] Yamaguchi K. Subramanian A. R. (2000). The plastid ribosomal proteins. Identification of all the proteins in the 50 S subunit of an organelle ribosome (chloroplast). *J. Biol. Chem.* 275 28466–28482. 10.1074/jbc.M005012200 10874046

[B55] Yamaguchi K. von Knoblauch K. Subramanian A. R. (2000). The plastid ribosomal proteins. Identification of all the proteins in the 30 S subunit of an organelle ribosome (chloroplast). *J. Biol. Chem.* 275 28455–28465. 10.1074/jbc.M004350200 10874039

[B56] Yoo S. D. Cho Y. H. Sheen J. (2007). Arabidopsis mesophyll protoplasts: a versatile cell system for transient gene expression analysis. *Nat. Protoc.* 2 1565–1572. 10.1038/nprot.2007.199 17585298

[B57] Yu F. Liu X. Alsheikh M. Park S. Rodermel S. (2008). Mutations in SUPPRESSOR OF VARIEGATION1, a factor required for normal chloroplast translation, suppress var2-mediated leaf variegation in Arabidopsis. *Plant Cell* 20 1786–1804. 10.1105/tpc.107.054965 18599582 PMC2518225

[B58] Yu F. Park S. Rodermel S. R. (2004). The Arabidopsis FtsH metalloprotease gene family: interchangeability of subunits in chloroplast oligomeric complexes. *Plant J.* 37 864–876. 10.1111/j.1365-313X.2003.02014.x 14996218

[B59] Zaltsman A. Ori N. Adam Z. (2005). Two types of FtsH protease subunits are required for chloroplast biogenesis and Photosystem II repair in Arabidopsis. *Plant Cell* 17 2782–2790. 10.1105/tpc.105.035071 16126834 PMC1242272

[B60] Zheng M. Liu X. Liang S. Fu S. Qi Y. Zhao J. (2016). Chloroplast translation initiation factors regulate leaf variegation and development. *Plant Physiol.* 172 1117–1130. 10.1104/pp.15.02040 27535792 PMC5047069

