# Supplementary figures and images for: Chloroplast Translation Elongation Factor EF-Tu/SVR11 Is Involved in var2-Mediated Leaf Variegation and Leaf Development in Arabidopsis
Source: Front Plant Sci. 2019 Mar 12;10:295. doi: 10.3389/fpls.2019.00295 (PMC6423176; doi:10.3389/fpls.2019.00295)

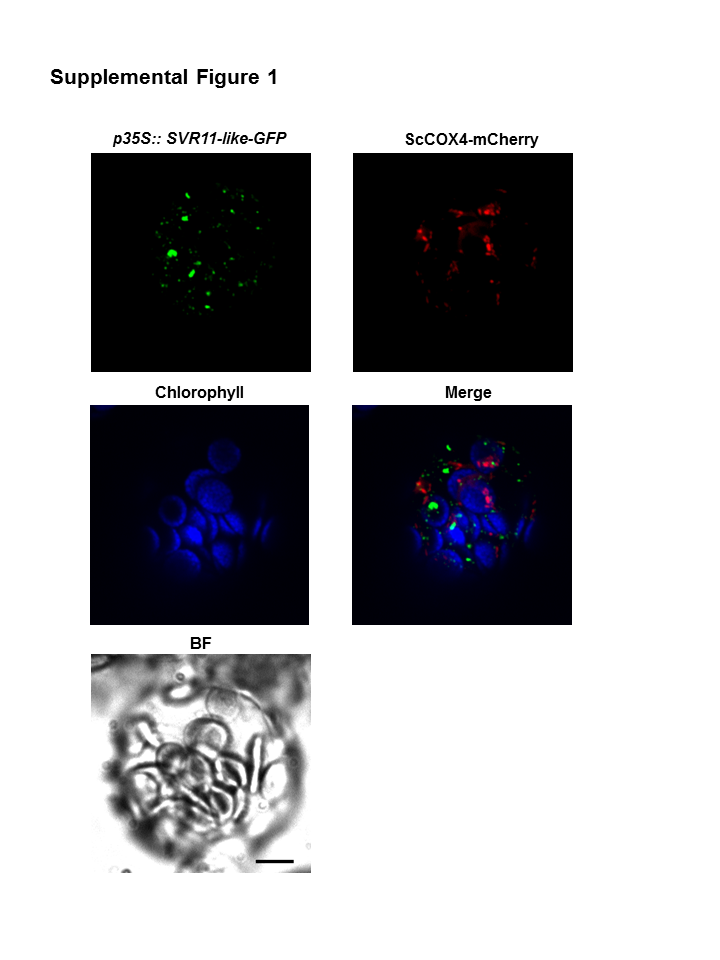

Supplement: FIGURE S1 — Transient expression of p35S::SVR11-like-GFP fusion protein in leaf protoplasts of plants expressing the mitochondrion marker ScCOX4-mCherry. Representative images of a single protoplast are shown. Bar stands for 10 μm. [file Image_1.TIF]

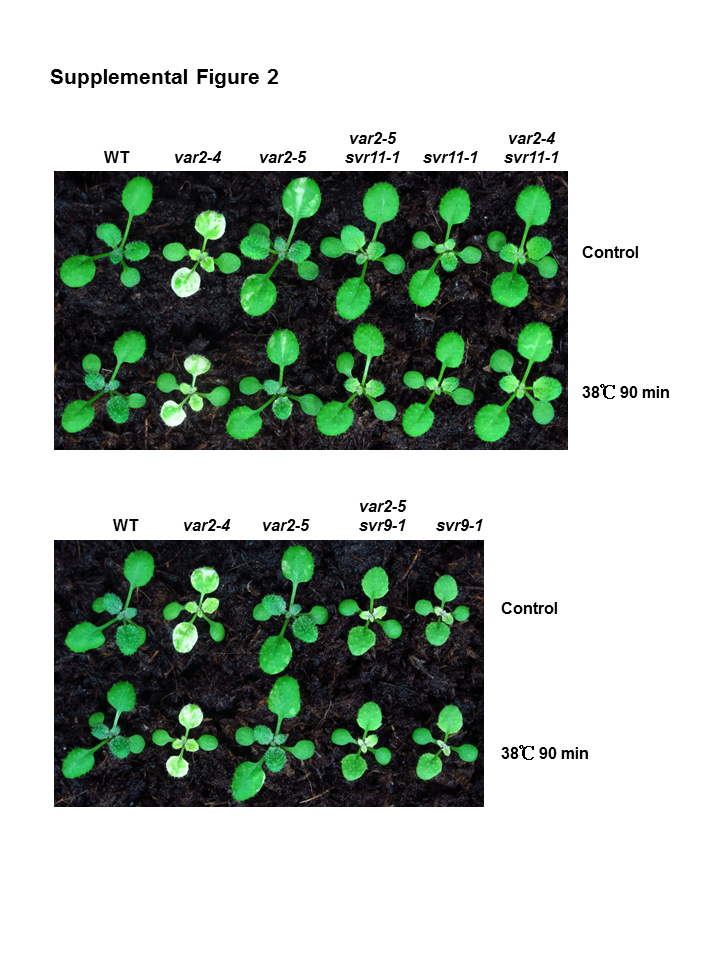

Supplement: FIGURE S2 — Representative seedlings treated with a moderate level heat stress. Eight-day-old seedlings grown at 22°C were treated at 38°C for 90 min, and then returned into 22°C for recovery. After 6 days recovery, seedlings were photographed. [file Image_2.TIF]

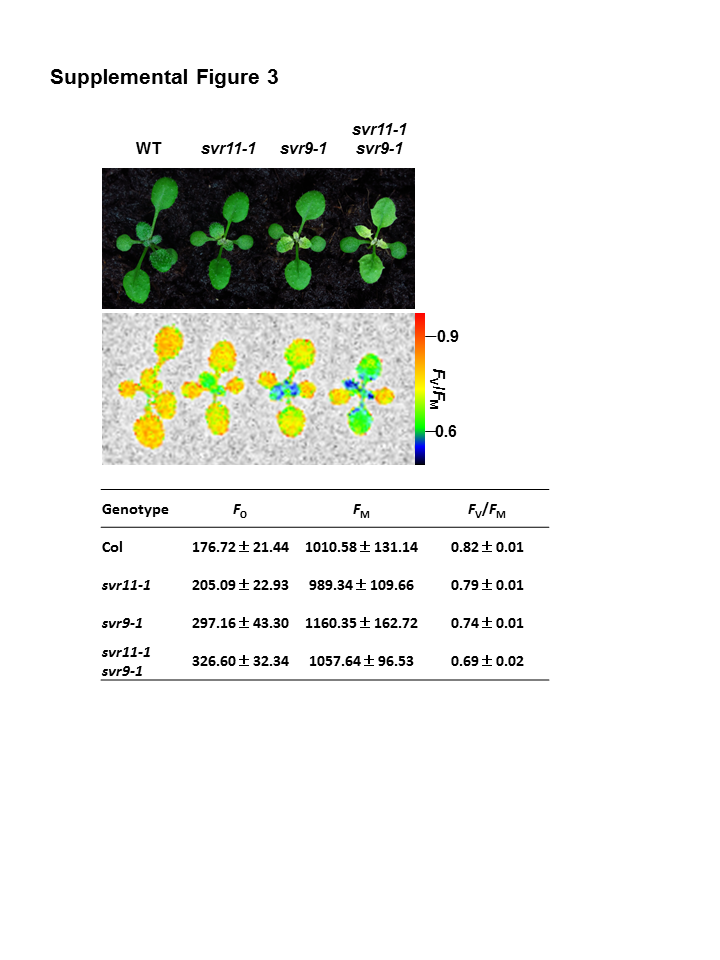

Supplement: FIGURE S3 — Representative 2-week-old seedlings and the corresponding FV/FM images of wild type, svr11-1, svr9-1, and the double mutant svr11-1 svr9-1. The average value of FO, FM, FV/FM were calculated from at least 10 individuals from each genotype. [file Image_3.TIF]
